# Congenital Desmoid Tumor of the Cheek: A Clinicopathological Case Report

**Published:** 2009-11-10

**Authors:** Amar Dalit, Meir Karen, Margulis Alexander

**Affiliations:** ^a^Department of Plastic and Reconstructive Surgery, Hadassah Medical Organization, Jerusalem, Israel; ^b^Department of Pathology, Hadassah Medical Organization, Jerusalem, Israel

## Abstract

**Objective:** Desmoid tumors are rare benign neoplasms of fibroblastic origin, frequently presenting in the abdomen of adult patients. Little is reported about clinical features and outcome of desmoid tumors appearing in infants and children, especially when they are located in the face. **Methods:** We report a girl with a desmoid tumor of her cheek, which was present at birth. **Results:** The tumor was treated by surgical excision, with no additional treatment, other than follow-up. No recurrence of the tumor was witnessed 15 months following surgical excision. **Summary:** Radical surgical treatment with tumor excision, sometimes accompanied by radiotherapy, is the current standard therapy for infantile desmoid tumors. Pharmacological treatment may be added in selected cases. These modalities may be challenging when desmoid tumors appear in the face because of risks of facial distortion and associated growth problems.

Desmoid tumors are benign fibrous neoplasms arising from the musculoaponeurotic structures throughout the body. This overgrowth of fibrous tissue is typically infiltrative, well-defined, and firm. Desmoid tumors are locally aggressive but lack the potential to metastasize.^1^ An aggressive clinical behavior with tendency for recurrence makes the treatment of these rare fibrous tumors challenging. Local recurrence rates after excision were reported to be as high as 70%.^2^ Complete surgical excision of desmoid tumors is believed to be the only effective method of cure by most authors.^1^

Common anatomic sites for appearance of desmoid tumors include extremities, abdominal wall, and mesentery. Desmoid tumors in the head and neck are uncommon in all age groups.

Infantile aggressive fibromatosis represents the childhood form of the musculoaponeurotic fibromatosis in adults.^2^ It arises as a solitary mass in skeletal muscle, adjacent fascia, or periosteum. Microscopically it exists in two forms: diffuse (mesenchymal) and desmoid. The less common desmoid form rarely occurs in infancy and even more rarely in the face. Little has been reported in the literature about the clinical features, the treatment, and the outcome of these lesions.

In this article, we report an infant girl with a desmoid tumor in the deep layers of her cheek. The clinical and imaging findings are followed by a histopathological description of the tumor.

## CASE REPORT

**Figure 1:**
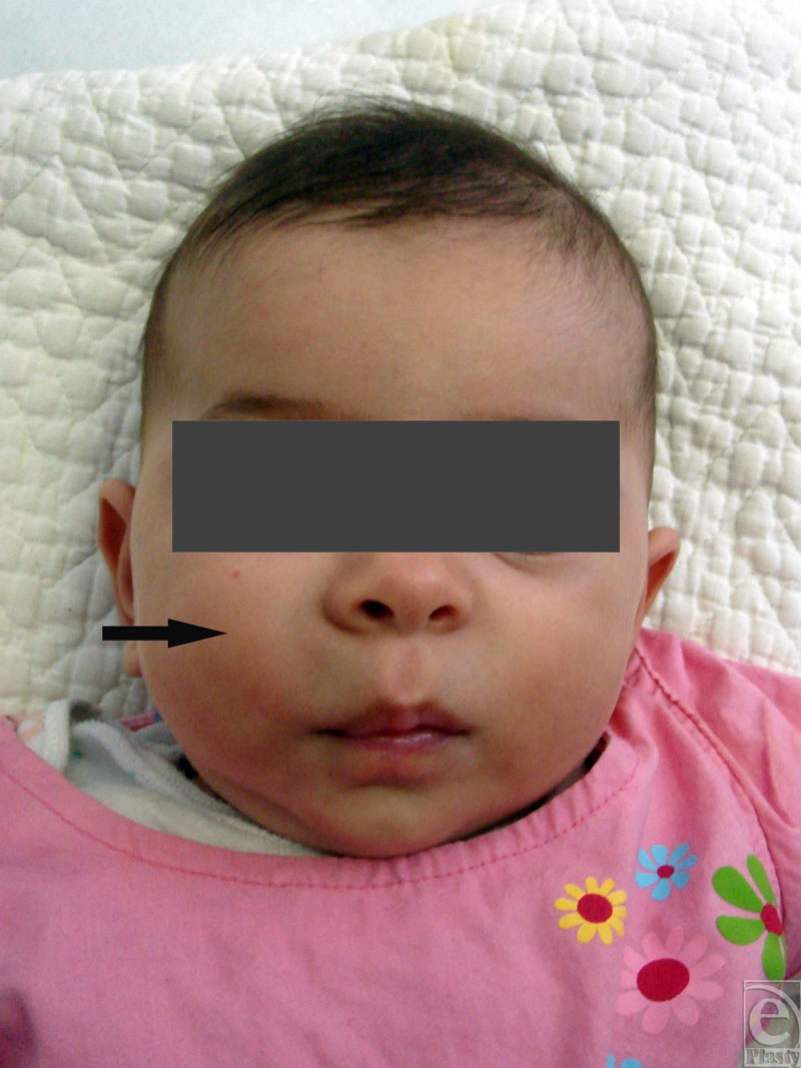
A 3-month-old girl with a solid, immobile mass in her right cheek. The lesion was present at birth and did not change dramatically in the first 3 months of life. The mass was firm on palpation, well-defined, and immobile (tumor is pointed out with an arrowhead).

A 3-month-old girl with a history of a solid, immobile mass in her right cheek presented to our clinic. The lesion was present at birth and did not change dramatically in the first 3 months of life. The mass was firm on palpation, well-defined, and mobile (Fig 1).

**Figure 2:**
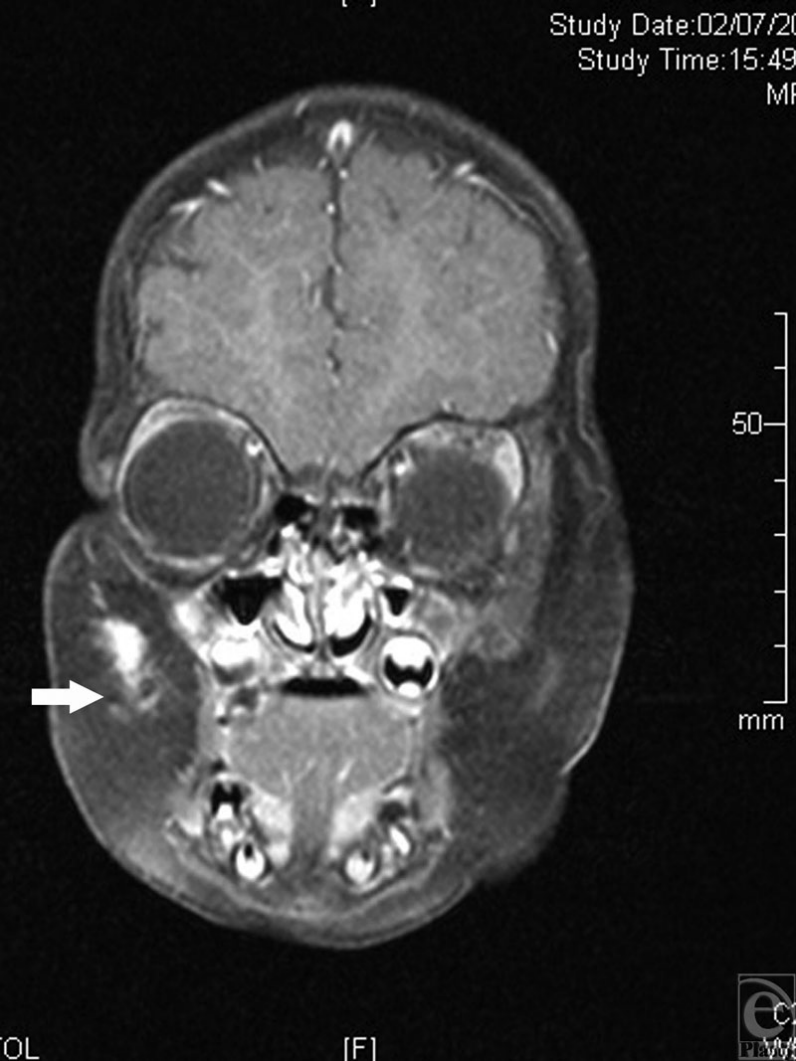
Magnetic resonance imaging study performed at the age of 3 months. A hyperintense fibrotic mass in the subcutaneous layers of the right cheek, penetrating to the upper lip is demonstrated (arrowhead).

Magnetic resonance imaging (MRI) study performed at this age demonstrated a hyperintense fibrotic mass in the subcutaneous layers of the right cheek, penetrating to the upper lip (Fig 2).

An incisional biopsy from the mass was performed. The histopathologic examination was nondiagnostic but was suspicious for a rhabdomyomatous mesenchymal hamartoma. With this working diagnosis, the patient was followed closely with a view to excision at a later age.

An additional MRI study performed, when the girl was 17 months old, demonstrated the same mass, which did not change in size or appearance from the previous MRI.

At this stage, a wide surgical excision of the mass was performed using the Weber-Fergusson approach. The skin above the mass was spared.

The gross pathological examination of the specimen revealed a solid, gray, ill-defined mass measuring 0.8 × 0.9 × 1.3 cm^3^. Microscopically, the lesion consisted of subcutaneous fat and muscle with an infiltrative process composed of haphazardly arranged bland spindle cells on a collagenous background, invading adjacent muscle fibers, and focally reaching the cauterized surgical margin. There was no necrosis, and no mitotic figures were observed. The immunohistochemical stain for β-catenin was positive in several nuclei. Focally, cells were also positive for α-actin. A desmin immunostain was negative. The findings were compatible with desmoid-type fibromatosis.

**Figure 3:**
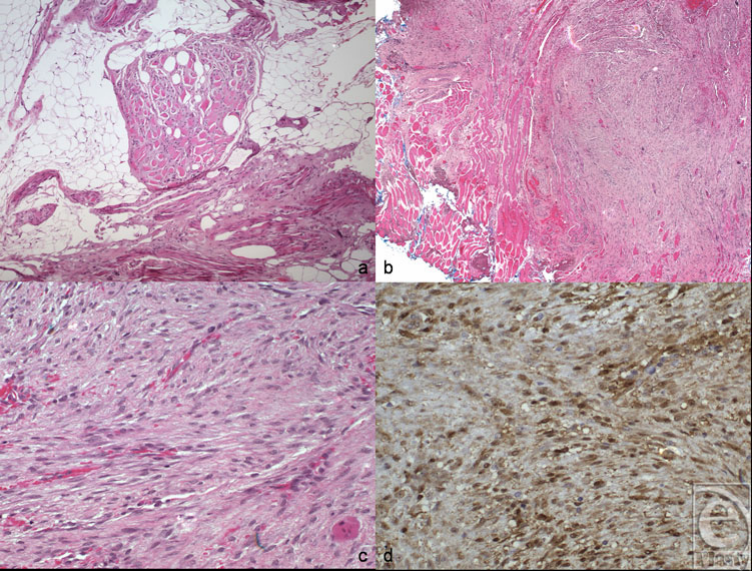
Histopathologic findings in desmoid type fibromatosis. (a) Preoperative biopsy specimen shows a mixture of adipose tissue and skeletal muscle infiltrated by collagen and bland round to spindle cells (hematoxylin and eosin, original magnification × 100). Findings from the surgical specimen (b-d). (b) Low-power magnification shows a spindle cell process infiltrating skeletal muscle (bottom left) and reaching the blue-inked surgical margin (top right) (hematoxylin and eosin, original magnification × 50). (c) High-power magnification shows a haphazardly arranged collagen bundles and spindle cells with round to oval nuclei. No mitotic figures and no necrosis seen (hematoxylin and eosin, original magnification × 200). (d) β-Catenin immunostain shows diffusely positive staining in tumor nuclei (immunoperoxidase, original magnification × 200).

The previous biopsy was reexamined, and the histopathological analysis was found to be compatible with an invading edge of fibromatosis (Fig 3).

The patient had no family history of Gardner syndrome or familial adenomatous polyposis. The patient was presented to the pediatric oncology tumor board. Because of the young age and the problematic location of the tumor, close clinical and radiological follow-up was recommended. A decision was made to avoid postoperative radiotherapy in this young girl because of the potential side effects of irradiation in this site and age.

**Figure 4:**
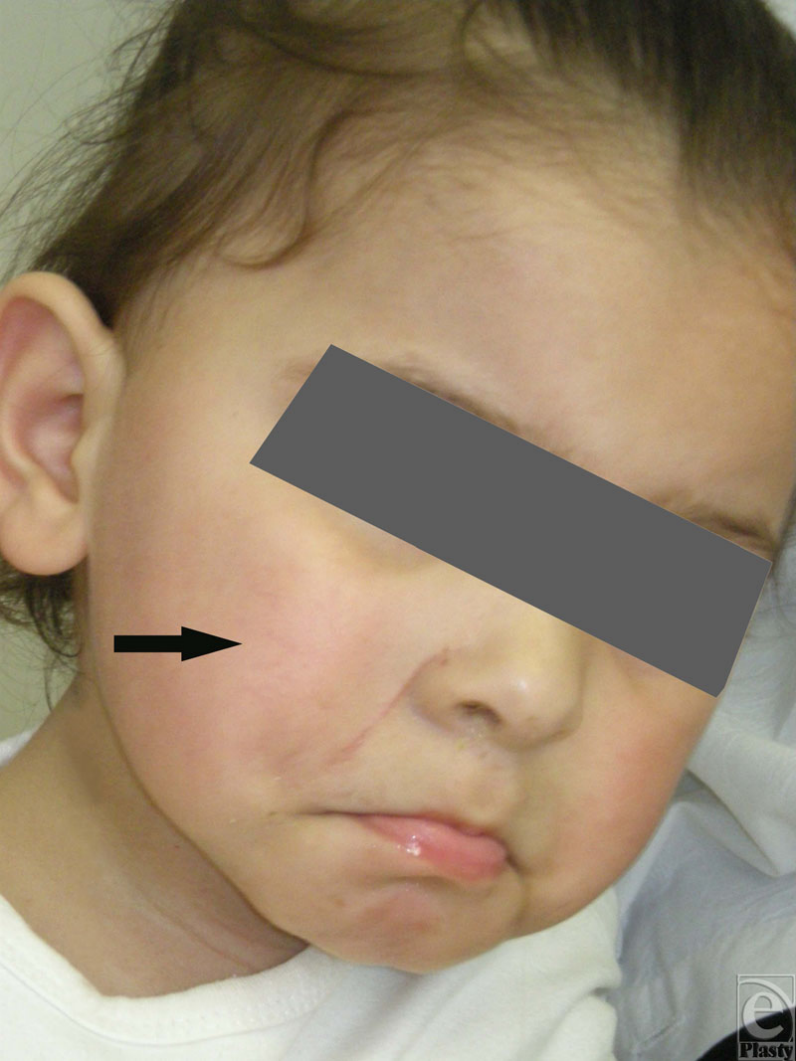
Postoperative follow-up photo at the age of 2 years. No recurrence of the tumor was witnessed 15 months after the surgical excision. Notice the well-appearing scar in the right nasolabial fold region.

No recurrence of the tumor was witnessed 15 months after the surgical excision (Fig 4).

## DISCUSSION

Desmoid tumors are histologically benign, fibrous neoplasms originating from the musculoaponeurotic structures of the body. They often appear as infiltrative, usually well-differentiated, firm overgrowths of fibrous tissue and tend to be aggressive locally.

Desmoid tumors are common in patients with familial adenomatous polyposis, an autosomal inherited disease caused by a genetic mutation in the *APC* (adenomatous polyposis coli) tumor suppressor gene, which is known to be a tumor suppressor gene. Desmoid tumors are associated with the bi-allelic *APC* mutation. One of the 2 known mutations occurs distal to the second β-catenin binding/degradation repeat of the gene.

Desmoid tumors are reported to account for 0.03% of all neoplasms.^1^ They are twice more common in females than in males and tend to occur in women after childbirth. In children, the female-to-male ratio is 1:1.

Although desmoid tumors are more common in the second to fifth decades of life, they can appear in younger children and older adults.

Infantile aggressive fibromatosis represents the childhood version of desmoid tumors seen in adults.

In the adult form, desmoid tumors most commonly develop in the anterior abdominal wall (arising from the rectus abdominis muscle) and in the shoulder girdle but may arise in any skeletal muscle. Retroperitoneal desmoids are common in familial *Polyposis coli* and Gardner syndrome and tend to be related to abdominal surgery.

In their subcutaneous locations, desmoid tumors are firm, smooth, and mobile. They often adhere to surrounding structures but usually spare the overlying skin. Desmoids were reported in intra-abdominal locations such as the urinary system. However, desmoid tumors are extremely rare in the head and neck, especially in infants and young children.

The pathogenesis of desmoid tumors is uncertain and may be related to trauma, hormonal factors, or mutation in the *APC* gene. Proliferative response of fibroblasts to estrogen was established.^3^

Computed tomographic (CT) scan and MRI are used for diagnosis and follow-up of desmoid tumors. MRI is better than CT scan in defining the pattern and the extent of involvement as well as recurrence after surgery.

Histologically, desmoid tumors are composed of abundant collagen surrounding poorly circumscribed bundles of spindle cells. These eosinophilic spindle cells contain regular nuclei and a pale cytoplasm. No mitoses or giant cells are present, a finding that helps differentiate these tumors from fibrosarcoma.^1^

Treatment of desmoid tumors consists of aggressive surgical resection with clear surgical margins. Positive margins after surgery can be associated with a high risk of recurrence (˜70%).^2^ Complete surgical excision of desmoid tumors is considered to be the only effective method of cure.

In patients who refuse surgery or represent poor surgical candidates, the following alternative modalities of treatment may be considered:

1. Radiation therapy can be considered for treatment of recurrent disease or as a primary modality used to avoid a mutilating surgical resection. Radiotherapy yields local control rates of approximately 75% to 80%.
2. Pharmacologic therapy with antiestrogen and prostaglandin inhibitors. The rationale behind this treatment is based on the possible endocrine etiology of desmoid tumors in which fibroblast proliferation is seen in response to estrogen. This hypothesis is supported by the fact that desmoid tumors most commonly appear in young women during or after pregnancy; the tumors regress during menopause, after tamoxifen treatment, and after exposure to oral contraceptives.
3. Chemotherapy (regimen of doxorubicin, dacarbazine, and carboplatin) is reserved mostly for recurrent extra-abdominal desmoid tumors.

Clinical features associated with high rate of recurrence include presentation at greater than 5 years of age, tumor location at the extremities, and an incomplete surgical resection.

Histological features associated with high rate of recurrence include microscopic evidence of tumor at the surgical margins, a mitotic index of 5 or more per 10 high-power fields, and evidence of necrosis and inflammation within the tumor.^4^

Infantile aggressive fibromatosis is a rare entity. It arises as a solitary mass in the skeletal muscle, adjacent fascia, aponeurosis, or periosteum. Microscopically it can present in 2 forms: the diffuse (mesenchymal) type and the desmoid type. The less common desmoid form is rarely seen in infancy. Several case reports have described an aggressive infantile fibromatosis involving the mandible.^5^–^12^

In most of these cases, radical surgical excision of the tumor and reconstruction of the mandible was the treatment modality, with no evidence of recurrence. However, in some cases, conservative surgical resection of the tumor was conducted with adjuvant chemotherapy and radiotherapy to minimize the recurrence rate and limit the surgical mutilation.^9^

Other case reports described desmoid tumor in the submandibular region of a 3-year-old girl^13^ and of an 18-month-old girl,^14^ the maxilla of a 7-year-old black Jamaican boy,^15^ base of the tongue,^16^ the parotid gland of a 7-year-old girl,^17^ the scalp,^18^ the nasal cavity and paranasal sinuses in a 2-year-old boy,^19^ and the base of skull (resulting in a fatal outcome) in a 10-year-old boy.^20^

A single-case report describing an extra-abdominal fibromatosis of the cheek^21^ suggests its exceptional location among the rare cases of desmoid tumors in the head and neck in infants and children.

A retrospective review dealing with treatment of desmoid tumors in the head and neck was recently published. Tostevin et al^22^ published data from 6 children with aggressive fibromatosis occurring in the head and neck. They concluded that primary excision is not always possible in this location, even though adjunctive modalities may not be feasible in children because of high rate of complications and interference with growth.^22^

The use of chemotherapy in infantile desmoid tumors was suggested in one study. The authors treated 3 children with progressive unresectable fibromatosis in unfavorable locations with pegylated liposomal doxorubicin (ie, nasal cavity, fossa infratemporalis, oral cavity, abdomen, and fossa supraclavicularis). Tumor response was obtained on MRI in all patients. All children have retained a normal cardiac function after the completion of chemotherapy as evaluated by left-ventricular shortening fraction. Severe neutropenia was not observed.^23^

## CONCLUSION

Desmoid tumors are benign, rare, firm fibrous neoplasms arising from the musculoaponeurotic structures throughout the body. These tumors are infiltrative and locally aggressive and thus tend to recur but lack the potential to metastasize. The pathogenesis of desmoid tumors is uncertain and may be related to trauma, hormonal factors, or a mutation in the *APC* gene. Desmoid tumors of the head and neck are uncommon in all age populations. Infantile aggressive fibromatosis is the childhood version of fibromatosis in adults. Complete surgical resection followed by radiation therapy is the current standard therapy. Pharmacological treatment may be added in selected cases.

Our report of an infant with a desmoid tumor on her cheek represents an example of the desmoid type of infantile aggressive fibromatosis arising in an exceedingly rare location. Surgical excision resulted in a complete removal of the tumor without evidence of local recurrence in 15 months' follow-up period.

## References

[1] Shields CJ, Winter DC, Kirwan WO, Redmond HP (2001). Desmoid tumors. Eur J Surg Oncol.

[2] Buitendijk S, van de Ven CP, Dumans TG (2005). Pediatric aggressive fibromatosis: a retrospective analysis of 13 patients and review of literature. Cancer.

[3] Raynham WH, Louw JH (1971). Desmoid tumors in familial polyposis of the colon. S Afr J Surg.

[4] Baerg J, Murph JJ, Magee JF (1999). Fibromatoses: clinical and pathological features suggestive of recurrence. J Pediatr Surg.

[5] Shira RB (1977). Aggressive juvenile fibromatosis involving the mandible: surgical excision with immediate reconstruction. Oral Surg Oral Med Oral Pathol.

[6] Carr RJ, Zaki GA, Leader MB, Langdon JD (1992). Infantile fibromatosis with involvement of the mandible. Br J Oral Maxillofac Surg.

[7] Sato K, Kawana M, Nonomura N, Takahashi S (2000). Desmoid-type infantile fibromatosis in the mandible: a case report. Am J Otolaryngol.

[8] Kutluhan A, Kiroglu AF, Ugras S (2002). Fibromatosis of the mandible in a child. Kulak Burun Bogaz Ihtis Derg.

[9] Seper L, Burger H, Vormoor J, Joos U, Kleinheinz J (2005). Aggressive fibromatosis involving the mandible-case report and review of the literature. Oral Surg Oral Med Oral Pathol Oral Radiol Endod.

[10] De Riu G, Meloni SM, Raho MT, Tullio A (2006). Complications of mandibular reconstruction in childhood: report of a case of juvenile aggressive fibromatosis. J Craniomaxillofac Surg.

[11] Jeblaoui Y, Bouguila J, Haddad S (2007). Mandibular aggressive fibromatosis. Rev Stomatol Chir Maxillofac.

[12] Krokidis M, Raissaki M, Mantadakis E (2008). Infantile fibromatosis of the mandible: a case report. Dentomaxillofac Radiol.

[13] Tagawa T, Ohse S, Hirano Y, Nomura J, Murata M (1989). Aggressive infantile fibromatosis of the submandibular region. Int J Oral Maxillofac Surg.

[14] Babin E, Goullet De Rugy M, Moreau S (2000). Head and neck desmoid tumor in children: a case report and review of the literature. Ann Otolaryngol Chir Cervicofac.

[15] Ogunsalu C, Barclay S (2005). Aggressive infantile (desmoid-type) fibromatosis of the maxilla: a case report and new classification. West Indian Med J.

[16] Shah AC, Katz RL (1988). Infantile aggressive fibromatosis of the base of the tongue. Otolaryngol Head Neck Surg.

[17] Ramanathan RC, Thomas JM (1997). Infantile (desmoid-type) fibromatosis of the parotid gland. J Laryngol Otol.

[18] Martinez-Lage JF, Acosta J, Sola J, Poza M (1996). Congenital desmoid tumor of the scalp: a histologically benign lesion with aggressive clinical behavior. Childs Nerv Syst.

[19] Mannan AA, Ray R, Sharma SC, Hatimota P (2004). Infantile fibromatosis of the nose and paranasal sinuses: report of a rare case and brief review of the literature. Ear Nose Throat J.

[20] Pulec JL (1993). Aggressive fibromatosis of the facial nerve. Ear Nose Throat J.

[21] Koeda S, Nagasaka H, Kumamoto H, Kawamura H (2005). Extra-abdominal fibromatosis of the cheek: report of a case. J Oral Maxillofac Surg.

[22] Tostevin PM, Wyatt M, Hosni A (2000). Six cases of fibromatosis of the head and neck in children. Int J Pediatr Otolaryngol.

[23] Wehl G, Rossler J, Otten JE (2004). Response of progressive fibromatosis to therapy with liposomal doxorubicin. Onkologie.

